# Anticancer Activities of *Polygonum odoratum* Lour.: A Systematic Review

**DOI:** 10.3389/fphar.2022.875016

**Published:** 2022-04-27

**Authors:** Thanut Khuayjarernpanishk, Sontaya Sookying, Acharaporn Duangjai, Surasak Saokaew, Asawadech Sanbua, Orapa Bunteong, Nutnicha Rungruangsri, Witchuda Suepsai, Patinya Sodsai, Jiraporn Soylaiad, Varintorn Nacharoen, Suwanna Noidamnoen, Pochamana Phisalprapa

**Affiliations:** ^1^ Chiwamitra Cancer Hospital, Ubon Ratchathani, Thailand; ^2^ Unit of Excellence on Clinical Outcomes Research and Integration (UNICORN), School of Pharmaceutical Sciences, University of Phayao, Phayao, Thailand; ^3^ Division of Pharmacy and Technology, Department of Pharmaceutical Care, School of Pharmaceutical Sciences, University of Phayao, Phayao, Thailand; ^4^ Center of Health Outcomes Research and Therapeutic Safety (Cohorts), School of Pharmaceutical Sciences, University of Phayao, Phayao, Thailand; ^5^ Department of Physiology, School of Medical Sciences, University of Phayao, Phayao, Thailand; ^6^ Division of Pharmacy Practice, Department of Pharmaceutical Care, School of Pharmaceutical Sciences, University of Phayao, Phayao, Thailand; ^7^ Division of Ambulatory Medicine, Department of Medicine, Faculty of Medicine Siriraj Hospital, Mahidol University, Bangkoknoi, Thailand

**Keywords:** cancer, *Persicaria odorata*, pharmacology, *Polygonum odoratum*, Vietnamese coriander

## Abstract

Cancers are a potential cause of death worldwide and represent a massive burden for healthcare systems. Treating cancers requires substantial resources, including skilled personnel, medications, instruments, and funds. Thus, developing cancer prevention and treatment measures is necessary for healthcare personnel and patients alike. *P. odoratum* (Polygonaceae family) is a plant used as a culinary ingredient. It exhibits several pharmacological activities, such as antibacterial, antifungal, antioxidant, anti-inflammatory, and anticancer. Several classes of phytochemical constituents of *P. odoratum* have been reported. The important ones might be polyphenol and flavonoid derivatives. In this systematic review, the activities of *P. odoratum* against cancerous cells were determined and summarized. Data were obtained through a systematic search of electronic databases (EMBASE, PubMed, Scopus, Thai Thesis Database, Science Direct and Clinical Key). Eight studies met the eligibility criteria. The cancerous cell lines used in the studies were lymphoma, leukemia, oral, lung, breast, colon, and liver cancer cells. Based on this review, *P. odoratum* extracts significantly affected Epstein-Barr virus (EBV) genome-carrying human lymphoblastoid (Raji), mouse lymphocytic leukemia (P388), human acute lymphocytic leukemia (Jurkat), breast adenocarcinoma (MCF-7), human colon adenocarcinoma (HT-29), human T lymphoblast (MOLT-4), human promyelocytic leukemia cell line (HL-60), human hepatocellular carcinoma (HepG2), and oral squamous cell carcinoma (SAS, SCC-9, HSC-3) through induction of cell apoptosis, arrest of the cell cycle, inhibition of cell proliferation, migration, and colonization. The molecular mechanism of *P. odoratum* against cancers was reported to involve suppressing essential proteins required for cell proliferation, colonization, migration, apoptosis, and angiogenesis. They were survivin, cyclin-D, cyclooxygenase 2 (COX-2), matrix metalloproteinase-9 (MMP-9), and vascular endothelial growth factor A (VEGF-A). The extract of *P. odoratum* was also involved in the protein kinase B (Akt)/mammalian target of rapamycin (mTOR) pathway by inhibiting the expression of Akt, phosphorylated Akt, mTOR, and phosphorylated mTOR. From the key results of this review, *P. odoratum* is a promising chemotherapy and chemopreventive agent. Further investigation of its pharmacological activity and mechanism of action should be conducted using standardized extracts. *In vivo* experiments and clinical trials are required to confirm the anticancer activity.

## Graphical Abstract

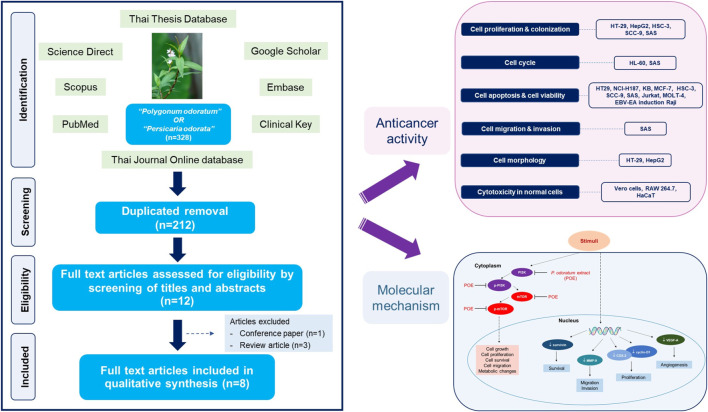

## Introduction

Cancers are a group of diseases that are one of the most common causes of death in every country worldwide. The prevalence of cancer worldwide is increasing dramatically. In 2020, the estimated number of new cases in 135 countries was 19.3 million, and cancer mortality was almost 10.0 million (Sung et al., 2021). Cancers have a high impact on national economies and healthcare systems. Cancer preventive measures and treatments are vital for medical and pharmaceutical practitioners. Sustainable and transitioning offers are sought for global cancer control. Among these measures, alternative medicines offer novel approaches. Traditional Thai medicine is a very important alternative medicine.

Medicinal plants have been widely used for a long time in traditional medicine. Patients accept herbal medicines because of their safety, efficacy, availability, and price. Identifying natural products as sources of anticancer drugs is extremely interesting.

*P. odoratum* Lour. (Polygonaceae family), or Vietnamese coriander, is a local vegetable indigenous to tropical Southeast Asia and is commonly grown in all regions of Thailand. The name was reclassified as *P. odorata* (Lour.) Sojak. The plant is always used as a culinary spice. It is also an indigenous herb generally used as an antiflatulence agent in traditional Thai medicine due to its pungent property. Several studies have reported diverse bioactive compounds and biological activities of *P. odoratum*. Its pharmacological activities have been documented and include antibacterial (Chansiw et al., 2019; Řebíčková et al., 2020), antifungal (Yanpirat and Vajrodaya, 2015), antioxidant (Somparn et al., 2013; Thongra-ar et al., 2021), anti-inflammatory (Okonogi et al., 2019), anti-osteoporosis (Sungkamanee et al., 2014) anticataractogenesis and antiretinopathy (Wattanathorn et al., 2017) and anticancer activities (Nanasombat and Teckchuen, 2009; Putthawan et al., 2017).

Many chemical compounds are found in *P. odoratum*, with more than 40 constituents identified in essential oil (Řebíčková et al., 2020). Typical organic compounds and aldehydes have been determined, for example, (Z)-3-hexenal, (Z)-3-hexenol, decanal, undecanal, dodecanal, 3-sulfanyl-hexanal, and 3-sulfanyl-hexan-1-ol (Starkenmann et al., 2006). The essential oil contains various terpenoids, especially sesquiterpenes. The most abundant compounds are dodecanal and decanal. Carboxylic acids and esters are present as minor traces in the oil (Hunter, 1996; Quynh et al., 2009).

Several constituents of in *P. odoratum* has been reported to be found in other plants and *Polygonum* sp. Among these compounds, alkaloids are very well known as anticancer agents. Furthermore, some of the rest compounds were reported about anticancer activities, for example, rutin, quercetin, and some of their biotransformed metabolites (Cipák et al., 2003; Araújo et al., 2013), tannins (Rajasekar et al., 2021; Tikoo et al., 2011; AlMalki et al., 2021; Youness et al., 2021), saponins (Elekofahinti et al., 2021), and quinones (Kuete et al., 2016; Shen et al., 2018).

The anticancer activities of *P. odoratum* have previously been investigated and reported. These studies used *in vitro* models and several cancer cell lines in their assays. However, the anticancer activities and underlying mechanisms have not yet been summarized. Therefore, the present work aimed to systematically review the activities of *P. odoratum* and its underlying mechanism against cancer. Eight articles were included into this systematic review. It was found that *P. odoratum* exhibited *in vitro* anticancer activity in several cell lines. Molecular mechanisms of the plant were investigated in one study. The proposed mechanisms were found to be involving in Akt/mTOR pathway, and the inhibitory effects through downregulation and reduction of key proteins which play the important roles in survival, migration and invasion, proliferation, and angiogenesis of cancer cells. The limitations of this systematic review were the number of the included studies, quantitative and qualitative analyses of phytochemical compounds, type of experiments, and number of studies that investigate the molecular mechanism. The suggestions for further study were also discussed herein.

## Methods

### Data Sources and Search Strategy

Two authors (SUS and SOS) independently searched electronic databases (EMBASE, PubMed, Scopus, Thai Thesis Database, Science Direct and Clinical Key). Relevant articles were searched from inception to March 2022. The strategic search terms used were “*P. odoratum*” or “*P. odorata.*” We also searched references in literature reviews and manuscripts published in journals. No limitations were placed on language and study design. In addition, we contacted related researchers and experts for details and explanations of the articles.

### Study Selection

After searching for articles, we removed duplicates, screened titles and abstracts, and obtained the full texts of each article. We included research classified as 1) studies of the anticancer activity of *P. odoratum* and 2) studies reporting measured outcomes (the anticancer effect). After the main search, a bibliographic search was performed to identify articles from conference proceedings for which the full text was available. We excluded articles in which the data had been obtained from prior studies. Accepted articles were included in this systematic review. Two investigators independently conducted the assessments.

### Outcome Measures

The primary outcomes of interest were measures of the anticancer effects of *P. odoratum*. The secondary outcomes were the molecular mechanisms of *P. odoratum* against cancer.

### Data Extraction

Two investigators independently reviewed each abstract and its associated full text. Each investigator also extracted data from each study for inclusion in the analysis. Data extraction was performed on study designs (part used, extract used, method and assay, outcomes). Discrepancies were resolved by consensus.

## Results

### Study Selection

**FIGURE 1:**
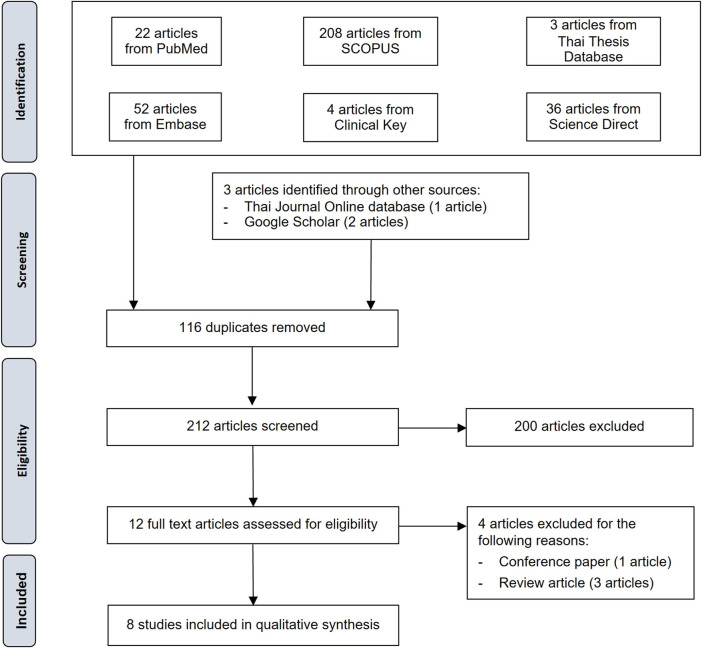
PRISMA flow diagram for the study selection process.

Three hundred twenty-five identified studies were systematically searched, and three studies were identified through other sources (1 from the Thai Journal Online database and 2 from Google Scholar). Two hundred twelve remained after duplicates were removed. After reviewing the information in the titles and abstracts, 200 studies were discarded. Of the 12 articles then assessed for eligibility, four were discarded (three review articles and one conference paper). The remaining eight studies were included in the qualitative analysis (Figure 1).

### Study Design

All included studies were *in vitro* studies. Their characteristics and main findings are summarized in Table 2. All were performed on different cancer cell lines: lymphoma, leukemia, T lymphoblast, oral, colon, breast, liver, and lung cancer. The plant parts used were the aerial part, leaf, stem, or leaf and stem, in the form of alcoholic extracts and alcoholic-based lyophilized powders. Four of eight studies reported results from phytochemical constituent analyses of the extracts. Screening tests of phytochemicals and LC-MS profiles of the methanolic extract were conducted by Devi Khwairakpam et al. (2019). However, this study did not identify any compounds in the LC-MS analysis. Wararatphoka et al. screened phytochemicals in the ethanolic leaf extract of *P. odoratum* (Woraratphoka et al., 2012). Identification and quantitative analysis of some flavonoids in methanolic leaf extract using HPLC techniques were performed by Nanasombat and Teckchuen (2009). The volatile oil composition used in the study by Kawaree et al. was identified and quantitated (Kawaree et al., 2006). Positive controls were considered in 4 studies. They were ellipticine and doxorubicin (Nanasombat and Teckchuen, 2009); catechin, trolox, and ascorbic acid (Woraratphoka et al., 2012); mitomycin (Putthawan et al., 2017); and vincristine (Semsri et al., 2018). The concentration of the extracts used in each assay varied.

The methods used in the studies were 12-*O*-hexadecanoylphorbol-13-acetate (HPA) induced- EBV-early antigen (EA) activation assay, 3-[4,5-dimethylthiazol-2-yl]-2,5 diphenyl tetrazolium bromide (MTT) assay, trypan blue assay, colorimetric cytotoxic assay using sulforhodamine B (SRB), propidium iodide (PI) flow cytometry, annexin V/PI fluorescein isothiocyanate (FITC) assay, agarose gel electrophoresis, sodium dodecyl sulfate-polyacrylamide gel electrophoresis (SDS-PAGE) and western blot, inverted light microscopy, and clonogenic and wound healing assay. The outcome measures were inhibitory effect, cell viability, cell proliferation, cell apoptosis, cell cycle phase, DNA ladder, cell morphology, colony formation, cell migration, key protein, and signaling molecule expression.

### *P. odoratum* Habit and Morphology

**FIGURE 2:**
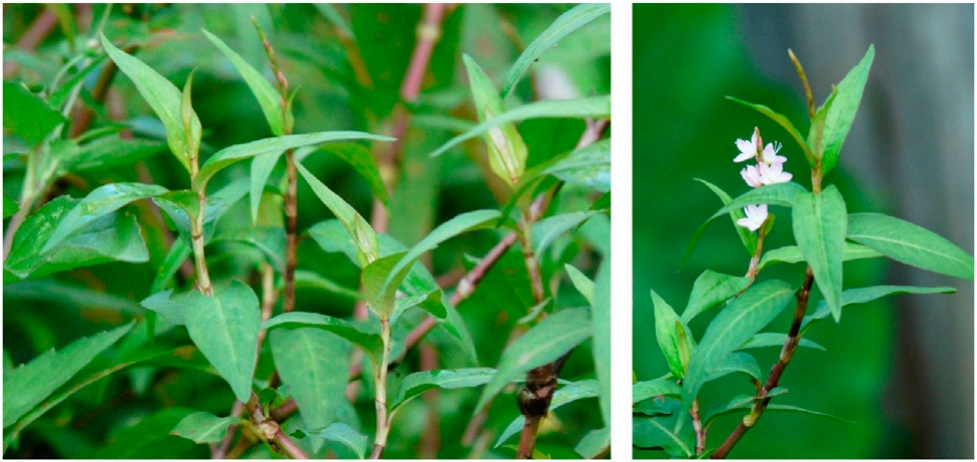
Characteristics of *P. odoratum* (adapted from Peterdehart3, 2021 and Kabilawan, 2014).

The plant is a perennial herb and is 15–30 cm tall. The stem is reddish-brown green in color and has a node at each leaf (Figure 2). The leaves are simple; are colored deep green; are lanceolate shaped and spirally arranged with thin white to brownish ochreate stipules; and have a strong smell when crushed. Inflorescences are in terminal positions with many small white, pink, and purple bisexual flowers, which bloom from October to December. Mature fruits are brown, simple, and dry indehiscent.

### Phytochemistry of *P. odoratum*

The chemical components of *P. odoratum* have been reported in several documents. Qualitative screening tests of methanolic extracts obtained from the aerial part of plants gave positive results for alkaloids, flavonoids, saponins, quinones, and glycosides, but negative results for steroids and tannins (Devi Khwairakpam et al., 2019). In a study by Nguyen et al., flavonoids, tannins, and triterpenoids were present in both ethanolic and aqueous extracts obtained from *P. odoratum* leaves. Saponins and alkaloids were not detected in ethanolic extracts, while coumarins and anthraquinones were not detected in aqueous extracts (Nguyen et al., 2020). Only volatile oil and tannins were detected in ethanolic leaf extracts by Woratatphoka et al. (2012).

The predominant phytochemical compounds found in extracts and essential oils obtained from *P. odoratum* are listed in Table 1. Most studies reported that C10 and C12 aldehydes and alcohols were the most abundant compounds in essential oil: decanal, dodecanal, decanol, and dodecanol. Other classes of compounds found in essential oil were primarily terpenoids. Some sesquiterpenes constituted in *P. odoratum* are β-caryophyllene, *cis*-caryophyllene, α-humulene, and α-selinene. Among the flavonoids found in the alcoholic extract of *P. odoratum* were rutin, catechin, quercetin, kaempferol, and isorhamnetin. Rutin was the most abundant flavonoid in *P. odoratum* leaf extract in a study by Nanasombat and Teckchuen (2009).

**TABLE 1:** Major phytochemicals found in the essential oil and extract obtained from *P. odoratum*.

Compound	Abundant (%)	References
C10 and C12 aldehydes and alcohols		
*N*-decanal	4.90–27.00 (essential oil)	Dũng et al. (1995), Hunter (1996), Kawaree et al. (2006), Quynh et al. (2009), Murray et al. (2019), Řebíčková et al. (2020)
*N*-dodecanal	31.40–57.55 (essential oil)	Dũng et al. (1995), Hunter (1996), Kawaree et al. (2006), Quynh et al. (2009), Murray et al. (2019), Řebíčková et al. (2020)
*N*-undecanal	0.30–1.83 (essential oil)	Dũng et al. (1995), Hunter (1996), Quynh et al. (2009), Murray et al. (2019), Řebíčková et al. (2020)
1-decanol	1.13–20.77 (essential oil)	Dũng et al. (1995), Hunter (1996), Quynh et al. (2009), Murray et al. (2019), Řebíčková et al. (2020)
1-dodecanol	3.30–11.4 (essential oil)	Dũng et al. (1995), Hunter (1996), Quynh et al. (2009), Murray et al. (2019), Řebíčková et al. (2020)
Alkanes		
*N*-undecane	1.30–2.52 (essential oil)	Dũng et al. (1995), Řebíčková et al. (2020)
Pentacosane	7.26 (essential oil)	Murray et al. (2019)
Sesquiterpenes and derivatives		
β-caryophyllene	0.00–36.5 (essential oil)	Dũng et al. (1995), Kawaree et al. (2006), Quynh et al. (2009), Řebíčková et al. (2020)
*Cis*-caryophyllene	0.20–3.88 (essential oil)	Dũng et al. (1995), Řebíčková et al. (2020)
α-humulene	0.27–4.50 (essential oil)	Dũng et al. (1995), Quynh et al. (2009), Murray et al. (2019), Řebíčková et al. (2020)
Caryophyllene oxide	1.42–8.20 (essential oil)	Dũng et al. (1995), Quynh et al. (2009), Řebíčková (2020)
Flavonoids and tannins		
Rutin	3.77 (methanolic leaf extract)	Nanasombat and Teckchuen (2009)
	0.04 (aqueous leaf extract)	Kawvised et al. (2021)
Ellagic acid	2.96 (ethanolic extract of aerial part)	Ahongshangbam et al. (2014)
	0.33 (aqueous leaf extract)	Kawvised et al. (2021)
Quercetin	0.08 (methanolic leaf extract)	Nanasombat and Teckchuen (2009)
	0.01 (aqueous leaf extract)	Ahongshangbam et al. (2014)
	2.67 (ethanolic extract of aerial part)	Kawvised et al. (2021)
Gallic acid	2.18 (ethanolic extract of aerial part)	Ahongshangbam et al. (2014)
	0.76 (aqueous leaf extract)	Kawvised et al. (2021)
Ferulic acid	1.30 (ethanolic extract of aerial part)	Ahongshangbam et al. (2014)
Chlorogenic acid	1.20 (ethanolic extract of aerial part)	Ahongshangbam et al. (2014)
	0.10–0.74 (methanolic leaf extract)	Pawłowska et al. (2020)
Apigenin	0.96 (ethanolic extract of aerial part)	Ahongshangbam et al. (2014)
P-coumaric acid	0.87 (ethanolic extract of aerial part)	Ahongshangbam et al. (2014)
Kaempferol	0.01(methanolic leaf extract)	Nanasombat and Teckchuen (2009)
	0.46 (ethanolic extract of aerial part)	Ahongshangbam et al. (2014)
Luteolin	0.20 (ethanolic extract of aerial part)	Ahongshangbam et al. (2014)
G-resorcyclic acid	0.05 (ethanolic extract of aerial part)	Ahongshangbam et al. (2014)
Catechin	0.34 (methanolic leaf extract)	Nanasombat and Teckchuen (2009)
	0.29–2.74 (methanolic leaf extract) 0.18 (aqueous leaf extract)	Pawłowska et al. (2020)
		Kawvised et al. (2021)
Isorhamnetin	0.01 (methanolic leaf extract)	Nanasombat and Teckchuen (2009)
Total phenolics		
Total phenolics	3.09 mg of GAE/g of fresh weight (aqueous extract)*	Zheng and Wang (2001)
	52.9 μmol catechin/g (fresh edible part)	Thu et al. (2004)
	52.00 µg gallic acid equivalents (GAE)/mg extract	Nanasombat and Teckchuen (2009)
	(dry methanolic leaf extract)	
	216.74 ± 15.33 µg GAE/mg extract (ethanolic based-lyophilized powder)	Woraratphoka et al. (2012)
	13.03 mg GAE/g of dry weight (aerial part)	Ahongshangbam et al. (2014)
	29.97 mg GAE/g extract (aqueous leaf extract)	Chansiw et al. (2019)
	52.59 mg GAE/g extract (methanolic leaf extract)	
	40.03 mg GAE/g extract (methanolic stem extract)	
	7.13–32.17 μg/g of dry matter (methanolic leaf extract)	Pawłowska et al. (2020)
	58.56 ± 3.86 µg GAE/mg extract (ethanolic leaf extract)	Nguyen et al. (2020)
	37.6 ± 1.59 µg GAE/mg extract (aqueous leaf extract)	
	223.00 ± 9.70 mg/ml GAE/mg extract (aqueous leaf extract)	Kawvised et al. (2021)

*Part used was not indicated.

**FIGURE 3:**
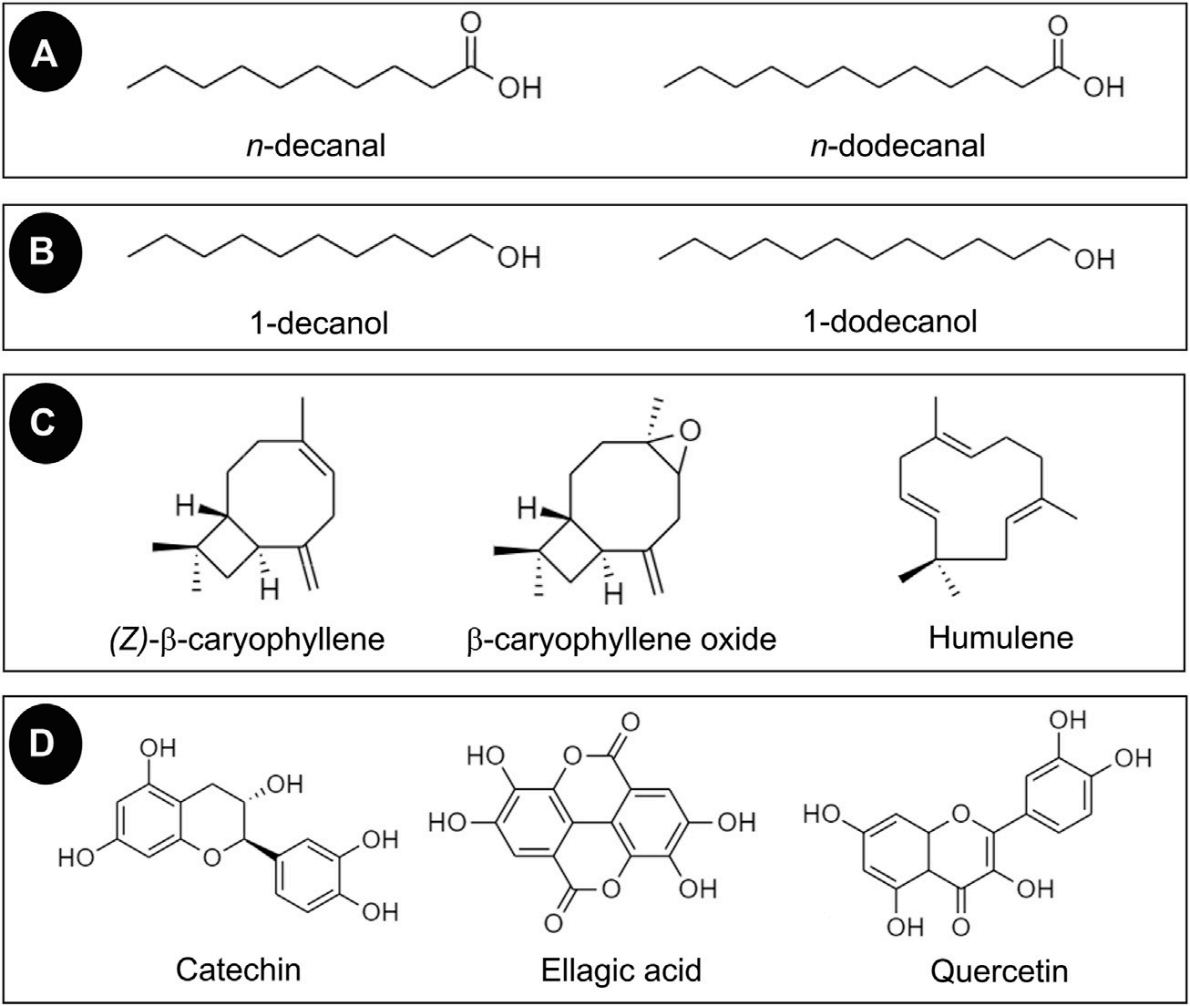
Chemical constituents found in *P. odoratum* classified as **(A)** aldehydes, **(B)** alcohols, **(C)** terpenoids, and **(D)** flavonoids and tannins.

In contrast, the compound or the free aglycone moiety was not detected in the leaf extract of *P. odoratum* in studies by Pawłowska et al. (2020) and Ahongshangbam et al. (2014), and only a small amount was detected in a study by Kawvised et al. (2021). Gallic acid was the predominant constituent in aqueous leaf extract (Kawvised et al., 2021). Tannins were not detected in a screening test by Devi Khwairakpam et al.; in contrast, another study reported a high abundance of this class of compounds (Kumar and Chaiyasut, 2017). Acids and esters were also found in *P. odoratum*. These were present as minor traces in the essential oil (Hunter, 1996) (Quynh et al., 2009). The total phenolic contents were determined in several studies. The structures of some chemicals found in *P. odoratum* are illustrated in Figure 3.

### Anticancer Activity of *P. odoratum*

A summary of the anticancer activities of *P. odoratum* on cancerous cell lines is presented in Table 2.

**TABLE 2:** *In vitro* anticancer activity of *P. odoratum*.

Author (year)	Part used	Extract	Cell line	Outcome	Method	Concentration (μg/ml)	Duration of incubation (hours)	Significant results*	Significant findings/remarks
Murakami et al. (1995)	N/A	Methanol (concentration not available)	EBVgenome-carrying human lymphoblastoid cell (Raji)	Cell viability	EA activation assay using HPA as an inducer	200	48	Inhibitory effect ≥70%	N/A
Kawaree (2006)	Leaf	Hydrodistillated volatile oil	Mouse lymphocytic leukemia (P388)	Cytotoxicity	Trypan blue assay	N/A	96	ED_50_ << 30 μg/ml	N/A
Nanasombat and Teckchuen (2009)	Leaf	Methanol (concentration not available)	Human oral epidermal carcinoma (KB)	Cell viability	Colorimetric cytotoxic assay using SRB	N/A	N/A	N/A	IC_50_ > 20 μg/ml
									Positive control:
									Ellipticine 0.61 ± 0.10 μg/ml
									Doxorubicin 0.18 ± 0.02 μg/ml
			Human breast adenocarcinoma (MCF-7)	Cell viability	Colorimetric cytotoxic assay using SRB	N/A	N/A	IC_50_ = 6.01 ± 0.08 μg/ml	Positive control:
									Ellipticine = 0.64 ± 0.16 μg/ml
									Doxorubicin = 0.23 ± 0.10 μg/ml
			Small cell lung carcinoma (NCI-H187)	Cell viability	MTT assay	N/A	N/A	N/A	IC_50_ > 20 μg/ml
									Positive control:
									Ellipticine = 0.60 ± 0.00 μg/ml
									Doxorubicin = 0.03 ± 0.00 μg/ml
			African green monkey kidney fibroblast (normal vero cell)	Cell viability	Colorimetric cytotoxic assay using SRB	N/A	72	N/A	IC_50_ > 50 μg/ml
									No toxic
Woraratphoka et al. (2012)	Leaf	70% ethanolic based-lyophilized powder	Human acute lymphocytic leukemia (Jurkat)	Cytotoxicity	MTT assay	N/A	48	IC_50_ = 146.80 μg/ml	Positive control:
									Catechin >400 μg/ml
									Trolox >400 μg/ml
									Ascorbic acid >400 μg/ml
			Human breast adenocarcinoma (MCF-7)	Cytotoxicity	MTT assay	N/A	48	N/A	IC_50_ = 205.20 μg/ml
									Positive control:
									Catechin >400 μg/ml
									Trolox >400 μg/ml
									Ascorbic acid = 78.60 μg/ml
			Human hepatocellular carcinoma (HepG2)	Cytotoxicity	MTT assay	N/A	48	N/A	IC_50_ > 400 μg/ml
									Positive control:
									Catechin >400 μg/ml
									Trolox >400 μg/ml
									Ascorbic acid >400 μg/ml
			Normal lymphocyte cells	Cytotoxicity	MTT assay	N/A	48	N/A	IC_50_ = 332.90 μg/ml
									Positive control:
									Catechin >400 μg/ml
									Trolox >400 μg/ml
									Ascorbic acid >400 μg/ml
Putthawan et al. (2017)	Leaf and stem	80% ethanol	Human colon adenocarcinoma (HT-29)	Cell proliferation	MTT assay	2000	24	Cytotoxic activity = 66.86% ± 12.95%	Positive control:
									Mitomycin C (50 μg/ml) = 17.32% ± 3.75%
				Cytotoxicity	MTT assay	250–4,000	N/A	N/A	CC_50_ = 775 μg/ml
				Apoptotic DNA ladder	Agarose gel electrophoresis	500, 1,000	48	N/A	Obvious DNA fragmentation were observed at 500, 1,000 μg/ml.
									Positive control:
									Mitomycin C (100 μg/ml)
									Dose dependent
									Quantitative data not available
				Cell morphology	Inverted light microscopy	500, 4,000	24	N/A	Early stage of apoptosis; cell shrinkage, denser cytoplasm and more tightly packed in shape were observed at 500 μg/ml.
									Loss of cell adhesion, reduced cell density, and membrane blebbing
									occurred at 4,000 μg/ml.
			Human hepatocellular carcinoma (HepG2)	Cell proliferation	MTT assay	2000	24	N/A	Cytotoxic activity = 68.94% ± 17.70%
									Positive control:
									Mitomycin C (50 μg/ml) = 81.35% ± 10.18%
				Cytotoxicity	MTT assay	250–4,000	N/A	N/A	CC_50_ = 1,665 μg/ml
				Apoptotic DNA ladder	Agarose gel electrophoresis	500, 1,000	48	N/A	Obvious DNA fragmentation observed at 500, 1,000 μg/ml
									Positive control:
									Mitomycin C (100 μg/ml)
									Dose dependent
									Quantitative data not available
				Cell morphology	Inverted light microscopy	500, 4,000	24	N/A	Cell shrinkage, denser cytoplasm and more tightly packed in shape were observed at 500 μg/ml.
									Loss of cell adhesion, reduced cell density, and membrane blebbing
									occurred at 4,000 μg/ml.
Semsri et al. (2018)	N/A	Ethanolic based-lyophilized powder	Human T lymphoblast (MOLT-4)	Cytotoxicity	MTT assay	15.625–500	48	IC_50_ = 56.1 ± 10.9 μg/ml	Positive control:
									Vincristine = 41.4% ± 9.8%
			African green monkey kidney fibroblast (normal vero cell)	Cytotoxicity	MTT assay	15.625–500	48	N/A	IC_50_ = 320.4 ± 13.1 μg/ml
			murine macrophage (RAW 264.7)	Cell viability	MTT assay	15.625–250	48	N/A	Cell viability = 97%, 86%, 78%, 74%, 32% (15.625, 31.25, 62.5, 125, 250 μg/ml)
									Dose dependent
Chansiw et al. (2018)	Leaf	95% methanol	Human promyelocytic leukemia cell line (HL-60)	Cell viability	MTT assay	50–1,000	48	IC_50_ = 350.00 ± 1.85 μg/ml	Dose dependent
								Cell viability ≈40%–70% (50, 100, 200, 500, 1,000 μg/ml)	
							72	IC_50_ = 38.00 ± 0.92 μg/ml	Dose dependent
								Cell viability ≈20%–40% (50, 100, 200, 500, 1,000 μg/ml)	
				Cell cycle phase	PI flow cytometric assay	50–200	48	N/A	G1-phase arrest
									Dose dependent
				Cell apoptosis	Fluorescent probe-based flow cytometric assay	50–200	48	Apoptosis ≈ 1%–7% (50, 100, 200 μg/ml)	Dose dependent
	Stem	95% methanol	Human promyelocytic leukemia cell line (HL-60)	Cell viability	MTT assay	50–1,000	48	IC_50_ = N/A	Dose dependent
								Cell viability ≈50% (1,000 μg/ml)	
							72	IC_50_ = N/A	Dose dependent
								Cell viability ≈20%–70% (500, 1,000 μg/ml)	
				Cell cycle phase	PI flow cytometric assay	50–200	48	N/A	G1-phase arrest
									Dose dependent
				Cell apoptosis	Fluorescent probe-based flow cytometric assay	50–200	48	Apoptosis ≈ 1%–3.5% (50, 100, 200 μg/ml)	
Devi Khwairakpam et al. (2019)	Arial part	70% methanolic based-lyophilized powder	Oral squamous cell carcinoma (SAS)	Cell proliferation	MTT assay	10–50	72	Proliferation ≈40%–80% (25, 50 μg/ml)	Dose dependent
				Cytotoxicity	PI flow cytometric assay	25–200	72	Cell death ≈60%–90% (100,150, 200 μg/ml)	Dose dependent
				Cell viability	Live/dead assay	20–80	48	Cell death ≈20%–40% (40, 80 μg/ml)	Dose dependent
				Cell cycle phase	PI flow cytometric assay	40–80	24	N/A	G2/M-phase arrest
									Dose dependent
				Cell apoptosis	Annexin V/PI FITC assay	100, 200	48	Apoptosis ≈ 3%–10% (100, 200 μg/ml)	Dose dependent
				Colony formation	Clonogenic assay	20–100	10–12 days after 24 h incubation	Survival fraction <0.4 (20, 40, 60, 80, 100 μg/ml)	Dose dependent
				Cell migration	Wound healing assay	10–50	24, 48, 72	Wound area ≈85%, 70%, 55% (10 μg/ml at 24, 48, 72 h)	Dose dependent and time dependent
								Wound area ≈90%, 80%, 50% (20 μg/ml at 24, 48, 72 h)	
								Wound area ≈90%, 80%, 75% (50 μg/ml at 24, 48, 72 h)	
				Key protein expression	SDS-PAGE and Western blot analysis	50–200	24	Fold change of cyclin D expression ≈0.2–0.4 (50, 100, 150, 200 μg/ml)	Dose dependent for all proteins
								Fold change of COX-2 expression ≈0.4–0.1 (50, 100, 150, 200 μg/ml)	
								Fold change of MMP-9 expression ≈0.7–1 (100, 150, 200 μg/ml)	
								Fold change of VEGF-A expression ≈0.6–0.8 (50, 100, 150, 200 μg/ml)	
								Fold change of survivin expression ≈0.2–0.6 (150, 200 μg/ml)	
				Akt/mTOR upstream signaling molecules expression	SDS-PAGE and Western blot analysis	50–200	24	Fold change of Akt-1 expression ≈0.1–0.4 (50, 100, 150, 200 μg/ml)	Dose dependent for all molecules
								Fold change of p-Akt (thr308) expression ≈0.2–0.4 (150, 200 μg/ml)	
								Fold change of p-Akt (Ser473) expression ≈0.2–0.5 (50, 100, 150, 200 μg/ml)	
								Fold change of mTOR expression ≈0.4–1 (50, 100, 150, 200 μg/ml)	
								Fold change of p-mTOR expression ≈0.1–0.2 (100, 150, 200 μg/ml)	
			Oral squamous cell carcinoma (SCC-9)	Cell proliferation	MTT assay	10–50	72	N/A	Proliferation >100% (10, 25, 50 μg/ml)
				Cytotoxicity	PI flow cytometric assay	25–200	72	Cell death ≈30%–60% (50, 100,150, 200 μg/ml)	Dose dependent
								Cell death ≈60% (μg/ml)	
			Oral squamous cell carcinoma (HSC-3)	Cell proliferation	MTT assay	10–50	72	Proliferation ≈80%–90% (10, 25 μg/ml)	Proliferation >100% (50 μg/ml)
				Cytotoxicity	PI flow cytometric assay	25–200	72	Cell death ≈20%–40% (25, 50, 100,150, 200 μg/ml)	Dose dependent
			Human keratinocyte (HaCaT)	Cell proliferation	MTT assay	10–50	72	Proliferation ≈80% (50 μg/ml)	Proliferation ≈90–100% (10, 25 μg/ml)
			(normal cell line)						Dose dependent

*Significant with *p* < 0.05 vs. control, N/A: data not available.

### Effects of *P. odoratum* in Cancer Cell Lines

#### Inhibition of Cell Proliferation and Colonization

Cell proliferation assays were performed on HT-29, HepG2, SAS, SCC-9, HSC-3, and HaCaT (normal cell line). The results were reported as cytotoxic activity in HT-29 and HepG2 cells. The value was 66.9% in HT-29 cells, which was much better than the 17.3% for the positive control treatment (50 μg/ml mitomycin). However, in HepG2 cells, cytotoxic activity was 68.9%, which was not as good as 81.3% for the positive control group. In OSCCs, the results were determined as percentage proliferation. In SAS cells, the extract of *P. odoratum* significantly reduced cell proliferation to 80% and 40% after 72 h of treatment at concentrations of 25 and 50 μg/ml, respectively. In HSC-3 cells, cell proliferation reduced to 90% and 80% at 10 and 25 μg/ml, respectively. The highest dose (50 μg/ml) did not affect cell proliferation. There was no significant result in SCC-9 cells. However, in the normal HaCat cell line, cell proliferation was reduced by 20% by the 50 μg/ml methanolic extract used in the same study.

A clonogenic assay was conducted in SAS cells. The survival fraction on days 10–12 after 24 h of incubation with the methanolic extract was significantly lower than that of the control (<0.4) in a dose-dependent manner (20–100 μg/ml).

#### Induction of Cell Cycle Arrest

To determine whether *P. odoratum* affected the cell cycle of cancer cells, each phase of the cycle was measured in a HL-60 and SAS cells using flow cytometry. Chansiw et al. found that HL-60 accumulated in the G1-phase after 48 h of incubation with methanolic leaf extract and methanolic stem extract of *P. odoratum* at 50–200 μg/ml. Nevertheless, no significant differences were observed. In SAS cells, G2/M phase arrest was observed 24 h after treatment with 40–80 μg/ml methanolic extract of the aerial part. The results were dose-dependent, and no significant effect was found.

#### Induction of Apoptosis and Reduction in Cell Viability

Cell viability, cytotoxicity, and apoptosis assays were carried out to determine apoptotic and cell death induction (Table 2).

Apoptosis tests were performed on HL-60 and SAS cells. The methanolic leaf extract induced a better apoptosis effect than the methanolic stem extract in HL-60. After 48 h of incubation with 50–200 μg/ml leaf and stem extracts, 1%–7% and 1%–3.5% apoptoses were observed, respectively. Both extracts induced cell apoptosis in a dose-dependent manner. In SAS cells, Devi Khwairakpam et al. discovered that 3%–10% apoptosis significantly occurred after incubating cells with 100 and 200 μg/ml methanolic extract for 48 h.

Nanasombat et al. observed cell viability tests in KB, MCF-7, NCI-H187, and African green monkey kidney fibroblast (normal Vero cell) using methanolic leaf extract. The results showed that the extract of *P. odoratum* moderately reduced the viability of MCF-7 cells with a 50% inhibitory concentration (IC_50_) of 6.01 μg/ml, compared with 0.64 and 0.23 μg/ml ellipticine and doxorubicin, respectively. No significance was found in KB, NCI-H187, and Vero cells. In HL-60, the methanolic leaf extract induced a better apoptosis effect than the stem extract, similar to the induction effects of apoptosis. Leaf extract at all concentrations (50–1,000 μg/ml) significantly resulted in cell viabilities of 20%–40% and 40%–70% after 48 and 72 h of incubation, respectively. The IC_50_ values of the leaf extract 48 and 72 h of treatment were 350 and 38 μg/ml, respectively. Stem extract reduced cell viability to 50% at 500 μg/ml after 48 h of incubation and to 20%–70% at 500–1,000 μg/ml after 72 h in a dose-dependent manner. In the study by Devi Khwairakpam et al., the methanolic extract obtained from the aerial part of *P. odoratum* induced cell death by 20% and 40% at 40 and 80 μg/ml, respectively, after 48 h of incubation. The inhibitory effect in cell viability test performed by Murakami et al. was reported as strongly active. The 200 μg/ml methanolic extract of *P. odoratum* showed the inhibitory effect of higher than 70% in HPA-mediated EBV-EA induction Raji cells.

Cytotoxicity tests were conducted in five studies. The 50% median effective dose (ED_50_) was determined using volatile oil from *P. odoratum* leaves in mouse lymphocytic leukemia (P388). The value was lower than 30 μg/ml after 96 h of incubation. The 70% ethanolic-based lyophilized preparation in the study by Woraratphoka et al. was tested for its IC_50_ in human acute lymphocytic leukemia (Jurkat), MCF-7, and HepG2 cells. The results were 146.80, 205.20, and >400 μg/ml, respectively. The extract exhibited moderate activity against Jurkat cell lines and low activity against MCF-7 and HepG2 cells. Ethanolic leaf and stem extracts were used in HT-29 and HepG2 cells by Putthawan et al. The 50% cytotoxic concentrations (CC_50_) were 775 and 1,665 μg/ml, respectively. No significance was observed in either cell line. MOLT-4 cells were used to investigate the IC_50_ of ethanolic-based lyophilized powder obtained from *P. odoratum*. The inhibitory effect was 56.10 μg/ml. The cytotoxicity of the methanolic extract of the aerial part on SAS, HSC-3, and SCC-9 cells was investigated using 25–200 μg/ml. After 72 h of treatment, cell death was significantly observed in a dose-dependent manner, with 20%–90%, 20%–40%, and 35%–60% in SAS, HSC-3, and SCC-9 cells at 25–200, 25–200, and 50–200 μg/ml, respectively.

#### Inhibition of Cell Migration and Invasion

The inhibition of cell migration and invasion was investigated in SAS cells by Devi Khwairakpam et al. using a wound healing assay. After incubation periods of 24, 48, and 72 h, the methanolic extract of *P. odoratum* at 10, 20, and 50 μg/ml showed significant inhibitory effects in dose- and time-dependent manners.

#### Alteration of Cell Morphology

Changes in cancer cell morphology were determined in HT-29 and HepG2 cells after 24 h of incubation with leaf and stem ethanolic extracts at 500–4,000 μg/ml. The results found in HT-29 cells were similar to those found in HepG2 cells. Cell shrinkage, denser cytoplasm, and tighter packing were observed at 500 μg/ml. These signs represent the early stage of apoptosis. Loss of cell adhesion, reduced cell density, and membrane blebbing occurred at 4,000 μg/ml.

### Molecular Mechanism of *P. odoratum* in the Process of Carcinogenesis

Two studies reported the effects of *P. odoratum* at the molecular level. Putthawan et al. performed apoptotic DNA ladder tests in HT-29 and HepG2 cells using 500 and 1,000 μg/ml ethanolic extracts. It was found that the *P. odoratum* extract at both concentrations induced DNA fragmentation in both cell lines; this was not observed in the control cell samples.

**FIGURE 4:**
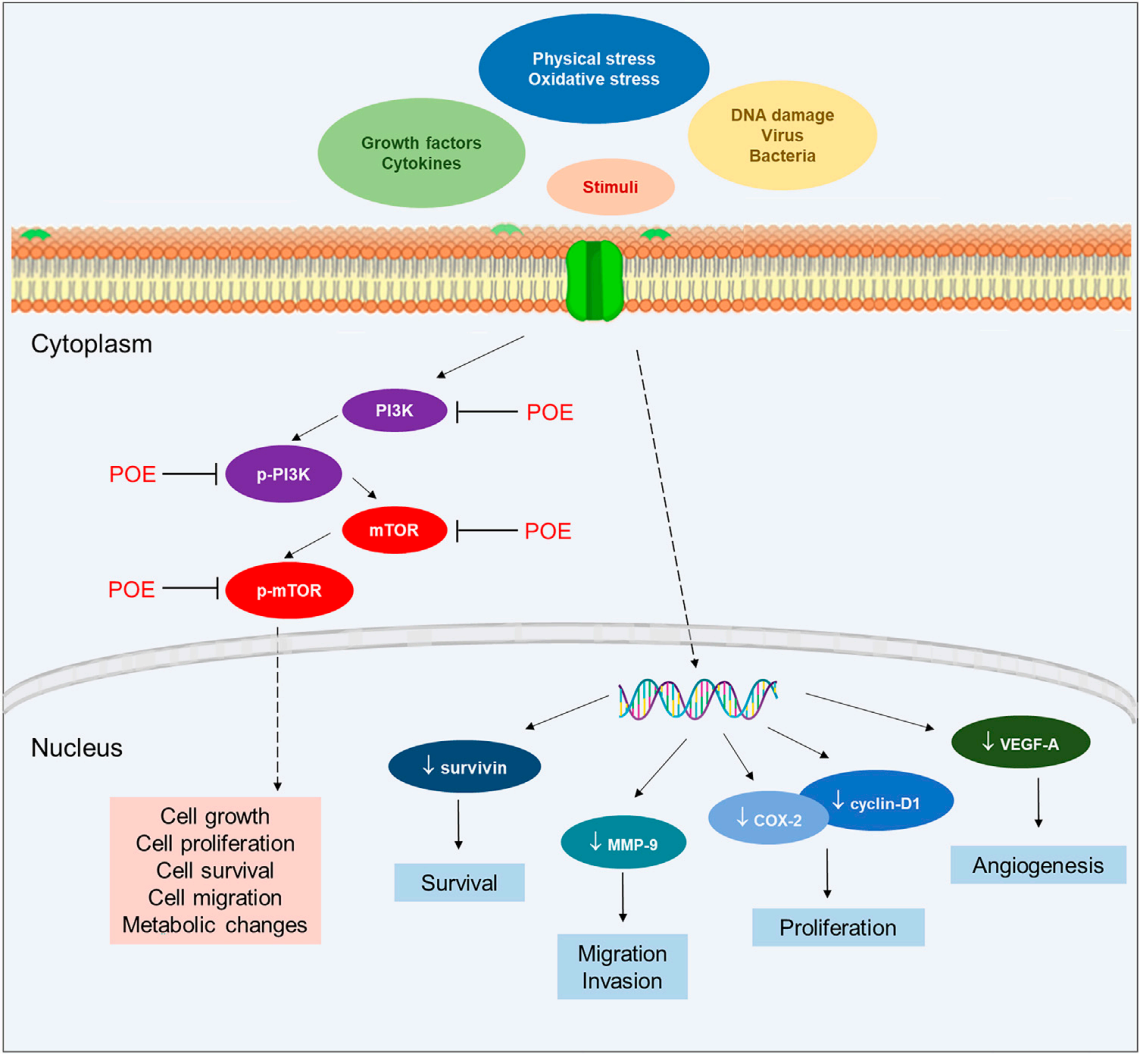
Molecular mechanism of *P. odoratum* methanolic extract (POE) as anticancer in oral squamous cell carcinoma, SAS cell line, investigated by Devi Khwairakpam et al. (2019). POE inhibited the expressions of survivin; a member of the inhibitor of the apoptosis family, VEGF-A; a vascular endothelial growth factor, which represents the essential roles in angiogenesis, and MMP-9; an enzyme involving in metastasis. POE also downregulated cyclin-D1; a proto-oncogene, which plays an important role in the regulation of cell cycle, and COX-2; a membrane glycoprotein which plays a vital role in the early stages of tumorigenesis. Besides, the extract downregulated the expression of PI3K and mTOR and reduced their phosphorylation. Thus, POE affected cell growth, cell proliferation, cell survival, cell migration and cell metabolism.

Another study reported on the mechanism of action of *P. odoratum* against cancer. The methanolic extract of the aerial part was used to explore the expression of key proteins and the regulation of critical signaling molecules in OSCC and SAS cell lines. The expression of key proteins (survivin, cyclin-D1, COX-2, VEGF-A, and MMP-9) decreased significantly when the dose increased. The fold changes of these proteins were in the range of 0.2–0.6, 0.2–0.4, 0.4–1, 0.6–0.8, and 0.7–1 for survivin, cyclin-D1, COX-2, VEGF-A, and MMP-9, respectively. Moreover, the extract suppressed signaling molecules in the Akt/mTOR pathway in a dose-dependent manner. They were Akt, phosphorylated Akt (Thr308, Ser473), mTOR, and phosphorylated mTOR. The changes in Akt and its phosphorylated forms, Thr308 and Ser473, were 0.1–0.4-fold, 0.2–0.4-fold, and 0.2–0.5-fold, respectively. The fold changes in mTOR and phosphorylated mTOR were 0.4–1 and 0.1–0.2, respectively. All results were dose-dependent. The molecular mechanism of action of *P. odoratum* is presented in Figure 4.

#### Cytotoxicity of *P. odoratum* on Normal Cell Lines

The cytotoxicity of *P. odoratum* in normal cells was demonstrated in four included studies. They were Vero cells, normal lymphocyte cells, murine macrophages (RAW 264.7), and HaCaT cells (Table 2). In normal Vero cells, methanolic leaf extract possessed low sensitivity, with an IC_50_ of >50 μg/ml, as lower concentrations were observed in cancerous cell lines in the study by Nanasombat et al. Another assay in Vero cells was carried out by Semsri et al. The IC_50_ was 320.4 μg/ml, which was much higher than the 56.1 μg/ml for MOLT-4 cells. In HaCaT cells, the methanolic extract of the aerial part at 50 μg/ml reduced cell proliferation by 20%. In other studies, Chan et al. revealed a CC_50_ of >100 μg/ml of *P. odoratum* leaf extract using various kinds of solvents in Vero cells (Chan et al., 2018). Cytotoxicity in RAW 264.7 cells was determined by Semsri et al. using ethanolic-based lyophilized powder of *P. odoratum*. The cell viabilities were 97%, 86%, 78%, 74%, and 32% after 48 h of incubation with 15.625, 31.25, 62.5, 125, and 250 μg/ml of sample, respectively. In addition, Chansiw et al. reported that 24 h of incubation with 200 μg/ml water leaf extract in RAW 264.7 cell lines resulted in a cell viability of approximately 80%. In comparison, approximately 10% reductions in cell viability were achieved with the same concentrations of water stem extract, dichloromethane leaf and stem extracts, and methanolic leaf and stem extracts (Chansiw et al., 2019). Kawvised et al. ascertained that the aqueous leaf extract of *P. odoratum* showed no toxicity. Moreover, the extract potentially attenuated the death of RAW 264.7 cells exposed to low-dose ionizing radiation (Kawvised et al., 2021).

## Discussion

In this systematic review, eight anticancer activities of *P. odoratum* are included. All were *in vitro* studies using several types of cancer cell lines (oral, lung, breast, colon, liver, T lymphoblast, lymphoma, and leukemia).

Cancer is a large group of diseases that can start in any organ or tissue, and they are ranked as a leading cause of death. They have a high impact on society and healthcare systems due to their economic toll and treatment and hospitalization costs. Cancers also seriously affect the quality of life of patients.

*P. odoratum* is an herb that belongs to the Polygonaceae family. It is also known as Vietnamese coriander or, in Thai, as “Phak Phai” and “Phak Paw.” The plant has long been used as a vegetable and is also used in traditional Thai medicine. Many lines of evidence have confirmed its pharmacological effects. Among these, its antioxidant and anticancer activities are of most interest. However, its anticancer activity has not yet been systematically summarized. This study aimed to review the anticancer activity of *P. odoratum* systematically.

The compounds reported as chemical constituents of *P. odoratum* are vastly diverse. The most abundant were C10–C12 aldehydes and alcohols. Moreover, flavonoids, terpenes, alkaloids, saponins, and tannins were detected in essential oil and extracts obtained from *P. odoratum*.

Eight studies included in this systematic review reported that the extracts of *P. odoratum* possessed significant anticancer activity against various types of cancerous cells. They are EBV-EA induction in Raji cells, P388, Jurkat, MCF-7, HT-29, MOLT-4, HL-60, HepG2, and SAS, SCC-9, HSC-3. Considering its activity against cancer, the chemical composition of *P. odoratum* is interesting.

A class of flavonoids was reported as abundant in *P. odoratum*, with rutin and its structure-related compounds, such as kaempferol and quercetin, predominant. Flavonoids are a group of polyphenolic compounds that have been reported to be responsible for the antioxidant, anti-inflammatory, and anticancer activities of plants (Yadav et al., 2010; González et al., 2011; Dias et al., 2021). Some flavonoids have been shown to regulate several proto-oncogenic signaling pathways (Satari et al., 2021). Rutin and quercetin had antioxidant effects in HepG2 cells (Alía et al., 2006; Kim and Jang, 2009). Rutin possessed anticancer activity in several cell lines when used as either a single therapy or with other antioxidants or herbal ingredients, such as quercetin and silibinin (Satari et al., 2021). Rutin, quercetin, and some of their biotransformed metabolites (rutin sulfate, methylquercetin, and quercetin glucuronide) showed pro-apoptotic and cytotoxic effects on HL-60 cells (Cipák et al., 2003; Araújo et al., 2013). A study by Iriti et al. found that rutin improved the anticancer activity of anticancer drugs and arrested the cell cycle in the G2/M and G0/G1 phases in MCF-7 cells (Iriti et al., 2017). The compound significantly inhibited the viability of the human lung cancer cell line (A549) and HT29 and attenuated superoxide production in HT29 cells. In addition, it affected the adhesion and migration of both cell lines (Sghaier et al., 2016). Quercetin showed significant anticancer activity in OSCC (SCC-25) through G1 phase arrest, mitochondria-mediated apoptosis, and decreased cell migration and invasion (Chen et al., 2013).

Tannins, a subclass of polyphenols, were detected in aqueous-ethanolic extracts and aqueous extracts of *P. odoratum* by Somparn et al. (2013) and Nguyen et al. (2020). A type of condensed tannin, catechin, was reported in several documents as a constituent of *P. odoratum* (Nanasombat and Teckchuen, 2009; Woraratphoka et al., 2012; Pawłowska et al., 2020; Kawvised et al., 2021) as well as its metabolite, gallic acid (Ahongshangbam et al., 2014; Kawvised et al., 2021). In a review by Rajasekar et al., it was found that tannins exhibited essential roles in the treatment of lung cancer through the induction of apoptosis and cell cycle arrest, attenuation of epithelial-to-mesenchymal transition, and regulation of tumor cell migration, invasiveness, and angiogenesis (Rajasekar et al., 2021). Many studies have revealed that tannins can enhance the anticancer effects of hormonal and chemotherapy medications, such as tamoxifen and doxorubicin (Tikoo et al., 2011; AlMalki et al., 2021; Youness et al., 2021). The molecular targets of tannic acid in anticancer activity were determined, and some results were consistent with those of Devi Khwairakpam et al. (Youness et al., 2021).

Saponins in the forms of triterpenoids and steroidal glycosides are gaining attention as promising anticancer agents. Many anticancer mechanisms of these compounds have been reported. They were found to have chemopreventive, cytotoxic, and antimetastatic activities and play a key role in multidrug-resistant cancers (Elekofahinti et al., 2021). In chemoprevention, saponins acted as anti-inflammatory agents. They also modulated the redox potential and arrested the cell cycle. The cytotoxic activity of saponins was described as apoptosis and autophagy induction. The metastasis of cancer cells was interrupted by saponins through anti-angiogenic activity and inhibition of cell adhesion molecules.

Alkaloids were reported to be found in *P. odoratum* by Devi Khwairakpam et al. and Nguyen et al.; however, identification of each compound is required (Devi Khwairakpam et al., 2019; Nguyen et al., 2020). In many studies, some alkaloids were detected in the genus *Polygonum,* such as *N*-*cis*-feruloyltyramine, *N*-*trans*-feruloyltyramine, and paprazine (Shen et al., 2018). Although this class of compounds is not a major constituent in *P. odoratum*, alkaloids are very well known for their cytotoxic and anticancer activities. Many conventional anticancer agents are naturally occurring alkaloid-derived compounds, for example, vincristine, vinblastine, camptothecin, and paclitaxel. Therefore, separation and identification of alkaloids are needed.

Quinones were also detected in *P. odoratum* extract, but no identification was reported, as with alkaloids (Devi Khwairakpam et al., 2019; Nguyen et al., 2020). Quinolones exhibited anticancer activities against several types of cancer cell lines. In a study of the cytotoxic effects of isolated quinones, it was found that quinones possessed satisfying activities in lung, liver, colon, and breast cancer cell lines (Kuete et al., 2016). Interestingly, some quinones provided IC_50_ values against cancers comparable to the cytotoxic drug doxorubicin. Chrysophanol, emodin, and plumbagin, all of which have been reported as quinones in *Polygonum* spp., showed IC_50_ values lower than 100 μM (Kuete et al., 2016) (Shen et al., 2018). Among these compounds, plumbagin exhibited significant anticancer activities in A549, colorectal adenocarcinoma (DLD-1), colorectal adenocarcinoma (Caco2), mesothelioma (SPC212), MCF-7, and HepG2, with an IC_50_ range of 0.06–1.14 μM, while doxorubicin provided 0.07–1.01 μM. Intriguingly, in the same experiment, plumbagin had lower cytotoxicity with normal fibroblast cells (CRL2120) than doxorubicin (67.7 vs. 0.59 μM, respectively). Plumbagin induced apoptosis in MCF-7 cells through the increased production of reactive oxygen species and the loss of MMP.

Many assays have confirmed that antiproliferative, antimigration, cell cycle arrest, and apoptotic effects of natural substances have anticancer activities. However, another mechanism against cancer might be the antioxidant effects of naturally occurring substances. Hence, the anticancer activity of *P. odoratum* might also be the result of its antioxidant effects, which have been reported by several studies (Chansiw et al., 2019; Nguyen et al., 2020; Zheng and Wang, 2001).

Different parts of *P. odoratum* and various solvents were used in the preparation methods of the included studies. Therefore, the phytochemical components of each extract might differ. In 2021, Azmi et al. reported that different phytochemical constituents were present in various parts of *P. odoratum* (Azmi et al., 2021). Moreover, the different extraction methods used by the studies produced diverse chemical profiles (Chansiw et al., 2019; Nguyen et al., 2020). In the study by Chansiw et al., the methanolic extract obtained from leaves was more potent in HL-60 cells than the extract from stems (Chansiw et al., 2018). The total phenolic and flavonoid yields obtained from leaves were higher than those obtained from stems in methanol, water, and dichloromethane extracts (Chansiw et al., 2019). Standardization could be implemented as a quality control measure.

Besides the quality control of natural products, the limitations of drugs from natural origins should be considered. The important one is the low bio-availability which lead to the requirement of high therapeutic dose. Nevertheless, the novel technology for drug delivery system is established. Nanoparticles was developed for drugs to reach target sites. Bhatnagar et al. demonstrated a safe and biocompatible method using bromelain nanoparticles to sustain release of the drug at the target site whilst also protecting the drug (Bhatnagar et al., 2015). *Antigono leptopus* containing-gold nanoparticles and *Acalypha indica* containing-copper oxide nanoparticles showed cytotoxicity against breast cancer cell lines (Balasubramani et al., 2015; Sivaraj et al., 2014). Therefore, this solution could be applied for further study.

Natural compounds were considered as anticancer drugs. They are also the potential adjuvants to cancer therapy. The combination chemotherapy were the new approaches to treat cancers. The administration of multiple chemotherapeutic drugs with different biochemical or molecular targets has attained numerous benefits like efficacy enhancement and amelioration of adverse effects. However, the risks from herb adverse effects and herb-drug interactions should be thoroughly considered.

## Conclusions and Future Recommendations

The anticancer activities of *P. odoratum* were investigated using *in vitro* experiments. Plant extracts showed significant activity against cell lines for leukemia, oral, lung, breast, colon, and liver cancer through induction of cell apoptosis, arrest of the cell cycle, inhibition of cell proliferation, migration, and colonization. The molecular mechanism of the inhibitory effects on the Akt/mTOR pathway was the same as the suppression of the critical proteins involved in cell survival, inflammation, proliferation, migration, apoptosis, and angiogenesis. (survivin, cyclin D, COX-2, MMP-9, and VEGF-A).

The limitations of this systematic review are:

1) A small number of studies met the criteria.
2) All studies were *in vitro-*based experiments.
3) The molecular mechanism was investigated by only two studies, with only one investigating apoptotic DNA fragmentation.
4) Screening tests of phytochemical constituents were reported by only three studies, while just one other study conducted quantitative analyses of some flavonoid compounds.

To get more information, further investigations of *P. odoratum* and its anticancer activity could be considered. Quantitative determination of phytochemicals and standardization of plant extracts should be considered as well as effective drug delivery systems. For *in vitro* experiments, standard positive controls should be used, as with normal control cell lines. *In vivo* experiments could also be performed.

## Data Availability

The original contributions presented in the study are included in the article/Supplementary Material, further inquiries can be directed to the corresponding authors.

## References

[B1] AlMalki F. M. Hassan A. M. Klaab Z. Abdulla S. Pizzi A. (2021). Tannin Nanoparticles (NP99) Enhances the Anticancer Effect of Tamoxifen on ER+ Breast Cancer Cells. J. Renew. Mater. 9 (12), 2077–2092. 10.32604/jrm.2021.016173

[B2] Ahongshangbam S. K. Shantibala Devi G. A. Chattopadhyay S. (2014). Bioactive Compounds and Antioxidant Activity of *Polygonum Odoratum* Lour. Int. J. Basic Appl. Biol. 2 (1), 94–97.

[B3] Alía M. Mateos R. Ramos S. Lecumberri E. Bravo L. Goya L. (2006). Influence of Quercetin and Rutin on Growth and Antioxidant Defense System of a Human Hepatoma Cell Line (HepG2). Eur. J. Nutr. 45, 19–28. 10.1007/s00394-005-0558-7 15782287

[B4] Araújo K. C. F. de M.B. Costa E. M. Valadares M. C. Oliveira V. (2013). Bioconversion of Quercetin and Rutin and the Cytotoxicity Activities of the Transformed Products. Food Chem. Toxicol. 51, 93–96. 10.1016/j.fct.2012.09.015 23000251

[B5] Azmi N. Zulkurnain E. I. Ramli S. James R. J. Halim H. (2021). The Phytochemical and Pharmacological Properties of Persicaria Odorata: A Review. Jpri 33 (41B), 262–279. 10.9734/JPRI/2021/v33i41B32366

[B6] Balasubramani G. Ramkumar R. Krishnaveni N. Pazhanimuthu A. Natarajan T. Sowmiya R. (2015). Structural Characterization, Antioxidant and Anticancer Properties of Gold Nanoparticles Synthesized from Leaf Extract(decoction)of Antigonon Leptopus Hook. &Arn. J. Trace Elem. Med. Biol. 30, 83–89. 10.1016/j.jtemb.2014.11.001 25432487

[B7] Ben Sghaier M. Pagano A. Mousslim M. Ammari Y. Kovacic H. Luis J. (2016). Rutin Inhibits Proliferation, Attenuates Superoxide Production and Decreases Adhesion and Migration of Human Cancerous Cells. Biomed. Pharmacother. 84, 1972–1978. 10.1016/j.biopha.2016.11.001 27829548

[B8] Bhatnagar P. Pant A. B. Shukla Y. Chaudhari B. Kumar P. Gupta K. C. (2015). Bromelain Nanoparticles Protect against 7,12-dimethylbenz[a]anthracene Induced Skin Carcinogenesis in Mouse Model. Eur. J. Pharm. Biopharm. 91, 35–46. 10.1016/j.ejpb.2015.01.015 25619920

[B9] Chan Y. S. Cheah Y. H. Chong P. Z. Khor H. L. Teh W. S. Khoo K. S. (2018). Antifungal and Cytotoxic Activities of Selected Medicinal Plants from Malaysia. Pak. J. Pharm. Sci. 31 (1), 119–127. 29348093

[B10] Chansiw N. Chotinantakul K. Srichairatanakool S. (2019). Anti-inflammatory and Antioxidant Activities of the Extracts from Leaves and Stems of *Polygonum Odoratum* Lour. Antiinflamm. Antiallergy. Agents Med. Chem. 18 (1), 45–54. 10.2174/1871523017666181109144548 30411695 PMC6446461

[B11] Chansiw N. Paradee N. Chotinantakul K. Srichairattanakool S. (2018). Anti-hemolytic, Antibacterial and Anti-cancer Activities of Methanolic Extracts from Leaves and Stems of *Polygonum Odoratum* . Asian Pac. J. Trop. Biomed. 8 (12), 580–585. 10.4103/2221-1691.248094

[B12] Chen S. F. Nien S. Wu C. H. Liu C. L. Chang Y. C. Lin Y. S. (2013). Reappraisal of the Anticancer Efficacy of Quercetin in Oral Cancer Cells. J. Chin. Med. Assoc. 76, 146–152. 10.1016/j.jcma.2012.11.008 23497967

[B13] Cipák L. Rauko P. Miadoková E. Cipáková I. Novotný L. (2003). Effects of Flavonoids on Cisplatin-Induced Apoptosis of HL-60 and L1210 Leukemia Cells. Leuk. Res. 27, 65–72. 10.1016/s0145-2126(02)00063-2 12479854

[B14] Devi Khwairakpam A. Monisha J. Roy N. K. Bordoloi D. Padmavathi G. Banik K. (2019). Vietnamese Coriander Inhibits Cell Proliferation, Survival and Migration via Suppression of Akt/mTOR Pathway in Oral Squamous Cell Carcinoma. J. Basic Clin. Physiol. Pharmacol. 31. 10.1515/jbcpp-2019-0162 31747377

[B15] Dias M. C. Pinto D. C. G. A. Silva A. M. S. (2021). Plant Flavonoids: Chemical Characteristics and Biological Activity. Molecules 26, 5377. 10.3390/molecules26175377 34500810 PMC8434187

[B16] Dũng N. X. Van Hac L. Leclercq P. A. (1995). Volatile Constituents of the Aerial Parts of VietnamesePolygonum odoratumL. J. Essent. Oil Res. 7 (3), 339–340. 10.1080/10412905.1995.9698534

[B17] Elekofehinti O. O. Iwaloye O. Olawale F. Ariyo E. O. (2021). Saponins in Cancer Treatment: Current Progress and Future Prospects. Pathophysiology 28, 250–272. 10.3390/pathophysiology28020017 35366261 PMC8830467

[B18] González R. Ballester I. López-Posadas R. Suárez M. D. Zarzuelo A. Martínez-Augustin O. (2011). Effects of Flavonoids and Other Polyphenols on Inflammation. Crit. Rev. Food Sci. Nutr. 51 (4), 331–362. 10.1080/10408390903584094 21432698

[B20] Hunter M. (1996). “Australian Kesom Oil—A New Essential Oil for the Flavour and Fragrance Industry,” in First Australian New Crops Conference, 8–11 July (The University of Queensland Gatton College). (Proceeding).

[B21] Iriti M. Kubina R. Cochis A. Sorrentino R. Varoni E. M. Kabała-Dzik A. (2017). Rutin, a Quercetin Glycoside, Restores Chemosensitivity in Human Breast Cancer Cells. Phytother. Res. 31 (10), 1529–1538. 10.1002/ptr.5878 28752532

[B22] Kabilawan S. (2014). Phak Phai Phak Paw. Available at http://2.bp.blogspot.com/-Do9tuYeydbI/U7eVerQpt5I/AAAAAAAAAAs/8n7ypRKSi8A/s1600/%E0%B8%9C%E0%B8%B1%E0%B8%81%E0%B9%84%E0%B8%9C%E0%B9%88.JPG.

[B23] Kawaree R. Phutdhawong W. Picha P. Ngamkham T. Chowwanapoonphon S. (2006). Chemical Compounds, Anticancer and Antioxidant Activity of Volatile Oil from *Piper Sarmentosum* Roxb., *Polyscias Fruticosa* Harms. And *Polygonum Odoratum* Lour. KMITL. Sci. J. 6 (2b), 499–504.

[B24] Kawvised S. Prabsattroo T. Munkong W. Pattum P. Iamsaard S. Boonsirichai K. (2021). *Polygonum Odoratum* Leaf Extract Attenuates Oxidative Stress and Cell Death of Raw 264.7 Cells Exposed to Low Dose Ionizing Radiation. J. Food Biochem. 0, 13909. 10.1111/jfbc.13909 34423456

[B25] Kim G. N. Jang H. D. (2009). Protective Mechanism of Quercetin and Rutin Using Glutathione Metabolism on HO-Induced Oxidative Stress in HepG2 Cells. Ann. N. Y Acad. Sci. 1171, 530–537. 10.1111/j.1749-6632.2009.04690.x 19723100

[B26] Kuete V. Omosa L. K. Tala V. R. Midiwo J. O. Mbaveng A. T. Swaleh S. (2016). Cytotoxicity of Plumbagin, Rapanone and 12 Other Naturally Occurring Quinones from Kenyan Flora towards Human Carcinoma Cells. BMC Pharmacol. Toxicol. 17 (60), 60. 10.1186/s40360-016-0104-7 27998305 PMC5175396

[B27] Kumar N. Chaiyasut C. (2017). Health Promotion Potential of Vegetables Cultivated in Northern Thailand: A Preliminary Screening of Tannin and Flavonoid Contents, 5α-Reductase Inhibition, Astringent Activity, and Antioxidant Activities. J. Evid. Based Complement. Altern Med 22 (4), 573–579. 10.1177/2156587216686689 PMC5871261 29228787

[B28] Murakami A. Jiwajinda S. Koshimizu K. Ohigashi H. (1995). Screening for *In Vitro* Anti-tumor Promoting Activities of Edible Plants from Thailand. Cancer Lett. 95 (1), 139–146. 10.1016/0304-3835(95)03879-2 7656222

[B29] Murray A. F. Satooka H. Shimizu K. Chavasiri W. Kubo I. (2019). *Polygonum Odoratum* Essential Oil Inhibits the Activity of Mushroom Derived Tyrosinase. Heliyon 5, e02817. 10.1016/j.heliyon.2019.e02817 31844734 PMC6895583

[B30] Nanasombat S. Teckchuen N. (2009). Antimicrobial, Antioxidant and Anticancer Activities of Thai Local Vegetables. J. Med. Plant Res. 3 (5), 443–339. 10.5897/JMPR.9000183

[B31] Nguyen V. T. Nguyen M. T. Nguyen N. Q. Truc T. T. (2020). Phytochemical Screening, Antioxidant Activities, Total Phenolics and Flavonoids Content of Leaves from *Persicaria Odorata* Polygonaceae. IOP Conf. Ser. Mater. Sci. Eng. 991, 012029. 10.1088/1757-899X/991/1/012029

[B32] Okonogi S. Kheawfu K. Holzer W. Unger F. M. Viernstein H. Mueller M. (2019). Anti-inflammatory Effects of Compounds from *Polygonum Odoratum* . Nat. Prod. Commun. 11 (11), 1651–1654. 10.1177/1934578x1601101107 30475499

[B33] Pawłowska K. A. Strawa J. Tomczyk M. Granica S. (2020). Changes in the Phenolic Contents and Composition of *Persicaria Odorata* Fresh and Dried Leaves. J. Food Compost. Anal. 91, 103507. 10.1016/j.jfca.2020.103507

[B34] Peterdehart3 (2021). Thai Herbs: Thai Herbs and Their Properties [Benefits of Phak Paw]. Available at https://lupuswiki.com/%E0%B8%9B%E0%B8%A3%E0%B8%B0%E0%B9%82%E0%B8%A2%E0%B8%8A%E0%B8%99%E0%B9%8C%E0%B8%82%E0%B8%AD%E0%B8%87%E0%B8%9C%E0%B8%B1%E0%B8%81%E0%B9%81%E0%B8%9E%E0%B8%A7/.

[B35] Putthawan P. Poeaim S. Areekul V. (2017). Cytotoxic Activity and Apoptotic Induction of Some Edible Thai Local Plant Extracts against Colon and Liver Cancer Cell Lines. Trop. J. Pharm. Res. 16 (12), 2927–2933. 10.4314/tjpr.v16i12.17

[B36] Quynh C. T. Iijima Y. Morimitsu Y. Kubota K. (2009). Aliphatic Aldehyde Reductase Activity Related to the Formation of Volatile Alcohols in Vietnamese Coriander Leaves. Biosci. Biotechnol. Biochem. 73 (3), 641–647. 10.1271/bbb.80709 19270388

[B37] Rajasekar N. Sivanantham A. Ravikumar V. Rajasekaran S. (2021). An Overview on the Role of Plant-Derived Tannins for the Treatment of Lung Cancer. Phytochemistry 188, 112799. 10.1016/j.phytochem.2021.112799 33975161

[B38] Řebíčková K. Bajer T. Šilha D. Houdková M. Ventura K. Bajerová P. (2020). Chemical Composition and Determination of the Antibacterial Activity of Essential Oils in Liquid and Vapor Phases Extracted from Two Different Southeast Asian Herbs-*Houttuynia Cordata* (Saururaceae) and *Persicaria Odorata (*Polygonaceae). Molecules 25, 2432. 10.3390/molecules25102432 PMC7287994 32456033

[B39] Satari A. Ghasemi S. Habtemariam S. Asgharian S. Lorigooini Z. (2021). Rutin: A Flavonoid as an Effective Sensitizer for Anticancer Therapy; Insights into Multifaceted Mechanisms and Applicability for Combination Therapy. Evid. Based. Complement. Alternat. Med. 2021, 9913179. 10.1155/2021/9913179 34484407 PMC8416379

[B40] Semsri S. Yongpisanpob N. Kongduang N. Homvisasevongsa S. Janwitayanuchit W. (2018). Biological Activities of *Basella alba, Polygonum Odoratum* and *Limnophila Geoffrayi* Bonati Extracts against Cancer and Phagocytosis of Macrophage. Huachiew Chalermprakiet Sci. Technol. J. 4 (2). (Article in Thai).

[B41] Shen B. B. Yang Y. Yasamin S. Liang N. Su W. Chen S. (2018). Analysis of the Phytochemistry and Bioactivity of the Genus Polygonum of Polygonaceae. Digit. Chin. med. 9 (1), 9–36. 10.1016/S2589-3777(19)30005-9

[B42] Sivaraj R. Rahman P. K. Rajiv P. Narendhran S. Venckatesh R. (2014). Biosynthesis and Characterization of Acalypha indica Mediated Copper Oxide Nanoparticles and Evaluation of its Antimicrobial and Anticancer Activity. Spectrochim Acta A. Mol. Biomol. Spectrosc. 129, 255–258. 10.1016/j.saa.2014.03.027 24747845

[B43] Somparn N. Jitvaropas R. Saenthaweesuk S. (2013). Hepatoprotective and Antioxidant Effects of *Polygonum Odoratum* L. Extract against Acetaminophen-Induced Hepatotoxicity in Rats. Thammasat Med. J. 13 (4), 456–464.

[B44] Starkenmann C. Luca L. Niclass Y. Praz E. Roguet D. (2006). Comparison of Volatile Constituents of Persicaria odorata(Lour.) Soják (Polygonum Odoratum Lour.) and Persicaria Hydropiper L. Spach (Polygonum Hydropiper L.). J. Agric. Food Chem. 54, 3067–3071. 10.1021/jf0531611 16608232

[B45] Sung H. Ferlay J. Siegel R. L. Laversanne M. Soerjomataram I. Jemal A. (2021). Global Cancer Statistics 2020: GLOBOCAN Estimates of Incidence and Mortality Worldwide for 36 Cancers in 185 Countries. CA Cancer J. Clin. 71, 209–249. 10.3322/caac.21660 33538338

[B46] Sungkamanee S. Wattanathorn J. Muchimapura S. Thukham-Mee W. (2014). Antiosteoporotic Effect of Combined Extract ofMorus albaandPolygonum Odoratum. Oxidative Med. Cell Longevity 2014, 1–9. 10.1155/2014/579305 PMC4247956 25478061

[B47] Thongra-ar K. Rojsanga P. Chewchinda S. Mangmool S. Sithisarn P. (2021). Antioxidant, α-Glucosidases and α-Amylase Inhibitory Activities of Persicaria Odorata. Cmujns 20 (3), 1–14. 10.12982/CMUJNS.2021.051

[B48] Thu N. N. Sakurai C. Uto H. Van Chuyen N. Lien D. T. Yamamoto S. (2004). The Polyphenol Content and Antioxidant Activities of the Main Edible Vegetables in Northern Vietnam. J. Nutr. Sci. Vitaminol (Tokyo) 50, 203–210. 10.3177/jnsv.50.203 15386933

[B49] Tikoo K. Sane M. S. Gupta C. (2011). Tannic Acid Ameliorates Doxorubicin-Induced Cardiotoxicity and Potentiates its Anti-cancer Activity: Potential Role of Tannins in Cancer Chemotherapy. Toxicol. Appl. Pharmacol. 251, 191–200. 10.1016/j.taap.2010.12.012 21194538

[B50] Wattanathorn J. Thiraphatthanavong P. Thukham-Mee W. Muchimapura S. Wannanond P. Tong-Un T. (2017). Anticataractogenesis and Antiretinopathy Effects of the Novel Protective Agent Containing the Combined Extract of Mango and Vietnamese Coriander in STZ-Diabetic Rats. Oxidative Med. Cell Longevity 2017, 1–13. 10.1155/2017/5290161 PMC5585686 28904737

[B51] Woraratphoka J. Intarapichet K. Indratapichate K. (2012). Antioxidant Activity and Cytotoxicity of Six Selected, Regional, Thai Vegetables. Am-euras. J. Toxicol. Sci. 4 (2), 108–117. 10.5829/idosi.aejts.2012.4.2.641

[B52] Yadav V. R. Prasad S. Sung B. Kannappan R. Aggarwal B. B. (2010). Targeting Inflammatory Pathways by Triterpenoids for Prevention and Treatment of Cancer. Toxins (Basel) 2, 2428–2466. 10.3390/toxins2102428 22069560 PMC3153165

[B53] Yanpirat P. Vajrodaya S. (2015). Antifungal Activity of Persicaria Odorata Extract against Anthracnose Caused by Colletotrichum Capsici and Colletotrichum Gloeosporioides. Malays. Appl. Biol. 44 (3), 69–74.

[B54] Youness R. Kamel R. A Elkasabgy N. Shao P. Farag M. (2021). Recent Advances in Tannic Acid (Gallotannin) Anticancer Activities and Drug Delivery Systems for Efficacy Improvement; A Comprehensive Review. Molecules 26, 1486. 10.3390/molecules26051486 33803294 PMC7967207

[B55] Zheng W. Wang S. Y. (2001). Antioxidant Activity and Phenolic Compounds in Selected Herbs. J. Agric. Food Chem. 49 (11), 5165–5170. 10.1021/jf010697n 11714298

